# Observation on “Disease”

**Published:** 1864-12

**Authors:** 


### Selections.

---

OBSERVATION ON "DISEASE."—BY J. A. A.—*Natura Sanat, Medicus Curat Morbos.*—WHAT IS DISEASE?—Disease is a change of structure in parts, tissues, organs or apparatuses of the system, varying them from the normal (physiologic) constitution.

This change may be in the ultimate moleculus only, (at "imperceptible distances"). As the normal metamorphoses of the material parts of the body develop the physiologic actions, or "functions," so do the abnormal metamorphoses develop pathological actions, or "symptoms." But the distinction is quite arbitrary—for as the greater part of the wonderful processes involved in LIFE, occur noiselessly, imperceptibly, and unconsciously, so may those of disease take place without "symptoms" of modified function before death, or apparent variation of the "phenomena of perceptible distance" after death.

Normal metamorphosis of the material gives healthy life—abnormal metamorphosis gives disease.

*An Idea to be Abandoned.*—That disease ever consists in "perversion of functions" without abnormal material change. The symptom "perversion of function" necessitates the presence of material change, visible or invisible, of the part involved—the remote cause of which may oftentimes be found in a distinct visible change in a more or less distant part, even when invisible in the organ whose function is perverted.

Whether the forces mainly impressing the body are "physical" or "vital," it is a plainly demonstrated natural law that their physiologic manifestation requires a physiologic material constitution. *Forces are never diseased!* Material often is.

*Paine and the Homœpathists*, respectively, attempt discussion of disease philosophically, and their treatment practically, by imputing everything of disease and everything of remedy to *forces*, nothing to the material through which, only, forces can be manifested. The former (with all Vitalists or Solidists) discusses disease as a metaphysical question, and attacks it, as Milton makes the fallen angels attack their unfallen brothers of incorporeal essence, with very solid cannon of ponderous mass and huge calibre, filled to the muzzle with very palpable gunpowder; whereas the Homœopathist, more consistently, attenuates the material of his attack infinitesimally, seeking thereby to get nearer the ultimate force with which he can reinforce and strengthen the errant force of organization. Hence Paine rattles dry *properties*, like peas in a basket, and the Homœopathist talks about "*potencies.*" *Par nobile fratrum.*

*General Considerotions.*—"Out of the fluid," says the elegant and exact Gerber,* "comes the solid, the shapen; all the parts of an animal body were once fluid, they have all been formed from the blood; and after death they will revert to the fluid state again. Organic matter, itself engendered and developed under the influence of the vital force, forms with water vivifying fluid of different kinds, either homogeneous and more or less consistent, or having numbers of extremely minute and regularly organized molecules mixed with it. This vivifying fluid has the faculty according to the circumstances in which it is placed, of coagulating or solidifying in different ways. The organic matter that is the most highly endowed with vitality separates in a state more or less highly organized, appropriately fashioned, and susceptible of life ever in conformity with the vitality and character of that which is around it, and with or without the solid elementary particles with which it is mingled; it then gradually acquires distinct and individual forms, which always bear appropriate relationship to the structures amidst which the separation takes place."

So far as physiology can inform us, the "solid and the shapen" come only from the blood and the lymph, or rather the portal blood, the chyle, and the lymph, all ultimating in the format'on of Blood, perfected by its final passage through the lungs, appearing in the aorta in its grade of highest vitality, or if you please, *capability* of highest vitality.

*Abstractly*, we must recognize the *formative force*, a phrase which is one of the landmarks of our present ignorance, to be trenched upon and removed further and further, as knowledge rescues more and more of science from the uncultivated domain

* Gerber's "General Anatomy, Text," p. 12.

of the unknown. It is this force which determines the forms assumed by species and individuals. Resident in the germ, a material substance, unknown, perhaps forever incomprehensible in its essence, nevertheless we know that its developmental energy can never be fully manifested, or even manifested at all, without appropriate supply of appropriate material. Nay, the very germ itself may be wholly blighted by imperfection of the material, or its future development arrested, variously deflected, or *diseased.* Without material pabulum, it may remain quiescent for ages. The kernel of wheat from the wrappings of a mummy, exhibits this force when furnished the material through which to operate, three thousand years after its fall from the parent stalk.

True, the phases of Force, Heat, Light, Chemical Affinity, etc., must be present, but these can only be evoked through the medium of matter arranged in peculiar forms, so that the proposition remains unchanged; without appropriate material there can be no manifestation of life—form or function.

With appropriate material there must always be disease. Nor is this a matter of merely speculative interest, as too many are disposed to imagine. Philosophically, it brings medicine into the domain of Physical Science; practically, it is hereby emancipated from the supremacy of visionary speculations and dogmas, mystery and charlatanry, into the freedom of a high and certain art. As there is seen something tangible to work upon, there is perceived something tangible to do.

*Physiology*, rightly understood, educes order and simplicity from the apparent chaos of manifestations of disease. In its light we are enabled to see that the systematic tables of diseases, with all the involved and inextricably confused attempts at classification, so painfully elaborated by the Nosologists, have no foundation in nature or fact. The doctrine of diseases as enticles which still infects some of our best professional minds, is departing to desuetude. Without becoming believers in the "unity of disease," we can cease to be "Ontologists," substituting for each dogma the solid substratum of physiologic law.

*The "Proximate Cause."*—The earlier Morbid Anatomists referred the cause of disease to the part showing morbid change in the "phenomena of perceptible distance," that is, to the part which showed change in its color, density, form, etc., but the evidences of modern physiology show that this is a great error. The real change may have been produced in a remote part which, on *post-mortem*, shows not the slightest visual evidence of lesion. "Morbid Anatomy," so-called, is fruitful, mainly in errors. It is subsidiary mainly to physiology, very little to pathology.

The essential changes of disease are molecular and cellular. The incidental—nay, *accidental*—changes are those which are perceptible on *post-mortem.*

The lungs may show "CONGESTION," but the *disease* be in the KIDNEYS!

Morbid Anatomy, so-called, might indicate that the subject died of Pneumonia, whereas, the real fact might be that he died of disease of the skin, the kidneys, the bowels, the brain, or spinal cord!

It will never do to base treatment on such fantasies as these. The real cause of death may be in the food, the air, the water, the stomach, the intestines, the adjuvant glandular tissues, the kidneys, or remote organs. Briefly, the *effect* on particular organs, which the Morbid Anatomist points out, so triumphantly, as illustrating his skill in diagnosis, is a proof of nothing but *ignorance.* You might as well go to the grave-yards, and vampire-like, dig up the bodies of the ancient dead and endeavor to show that each died of disease of the part which most speedily and completely showed decomposition.

Briefly, Morbid Anatomy, with its "beautiful illustrations," shows nothing, proves nothing, simply because the ultimate elements of diseases, like the ultimate changes of health, are to be found among molecules and cells, undiscoverable by aided or unaided vision, save in results.

The only approximation to certainty is gained by contrast of the physiological to the pathological. It is probable that the molecules and cells (inseparable in physiological relation,) develop in strict accordance with the conditions in which they are placed. In other words: The individual cell is the type of organic life, and in its changes every function of the most highly developed organization is expressed. To each there are definite conditions of existence in its typical form; that is to say, the cells developing the individuals of each type do so under certain circumstances, *change the conditions and*

*the cells develop different forms, tending to the type of those normally developed under similar conditions.*

When the transformation is consistent with the usual *functions* of the part, it is little noticed, but when inconsistent we recognize disease. That which is the natural or healthy form in one being, developed in another by the equable operation of the same all-pervading laws, involves impairment and even dissolution. Thus the causes of one existence determine cessation of another.

"The Universal Cause
Acts not by partial but by general laws."

We fail accurately to draw the line between health and disease, definitions are at fault, because the differences are but phases of normal organic life. It is, therefore, fitting to view the phenomena of disease with a single, unprejudiced eye; not as the product of marvelous or miraculous causes; not as the means of revenge of wrathful deities, as anciently believed and, even now tacitly admitted by the more ignorant and credulous; not as essential mysteries, locked up forever with the unapproachable arcana of Creative Intelligence, and hence to be combated, if at all, by marvels and miracles, by appeasing charms, by specifics and perturbating methods, by strange influences and fortuitous appliances, but rather as the orderly effect of forces as subject to observation and discoverable as gravitation and affinity, acting upon matter as tangible to examination as the elements of inorganic being, and therefore to be controlled, through the insight which science affords, with as much certainty as we can regulate the reaction of physical agencies upon inanimate objects.

Medicine should no longer be considered *asinus portans mysteria*, but as an eagle, with powerful wing and keen eye, facing unblenchingly the sun of Modern Science.—*Chicago Medical Journal.*

